# Qualitative and quantitative methods detection of SDS based on polyelectrolyte microcapsules

**DOI:** 10.1038/s41598-021-04343-z

**Published:** 2022-01-07

**Authors:** Aleksandr L. Kim, Egor V. Musin, Alexey V. Dubrovskii, Sergey A. Tikhonenko

**Affiliations:** Institute of Theoretical and Experimental Biophysics Russian Academy of Science, Institutskaya St., 3, Puschino, Moscow, Russia 142290

**Keywords:** Environmental chemistry, Freshwater ecology, Urban ecology, Analytical chemistry, Environmental chemistry, Supramolecular chemistry

## Abstract

Sodium dodecyl sulfate (SDS) is the most widely used anionic surfactant. Its frequent use causes environmental pollution and negative effects on living organisms (even at low concentrations ≈ 20 μg/ml). Thus, cheap and fast methods are needed to detect this surfactant in wastewater and surface waters in order to prevent the negative effects of SDS on the environment and human beings. We discovered that sodium dodecyl sulfate is capable of destroying polyelectrolyte microcapsules, which has been demonstrated by the number of sedimented polyelectrolyte microcapsules (PMC) before and after incubation in SDS solution. Therefore, it was proposed to use PMCs to create qualitative and quantitative diagnostic systems for the determination of SDS in solution. The qualitative system is a polyelectrolyte microcapsules containing polyallylamine labeled with a fluorescent dye—FITC. An excess SDS concentration of more than 5 μg/ml in the analyzed medium leads to the destruction of PMC and an increase in the fluorescence intensity of the solution, which is recorded by a fluorometer. The quantitative diagnostic system is based on turbidimetry of the PMC suspension before and after incubation in an anionic surfactant solution. This system has a range of detectable SDS concentrations from 10 to 50 μg/ml, with a standard deviation of no more than 11%.

## Introduction

Surface active agents (surfactants) are a group of chemicals that have a polar hydrophilic headgroup and a non-polar lipophilic hydrocarbon tail group^1^. This structure of surfactants allows them to be used in households and industries to increase the solubility of non-water-soluble substances, such as cleaning agents and emulsifiers. Global production of synthetic surfactants was 7.2 million tons in 2000^2^; since 2006, this value has risen to 12.5 million tons^3^ and these numbers will grow with the growth of the detergent and cosmetics industry. After use, the residual surfactants are discharged into the sewage system or directly into surface water, resulting in an increase in the level of surfactants in the environment and a significant impact on the ecosystem^1^.

The toxicity of surfactants to organisms is well known^4^ and depends on the physico-chemical properties of the surfactants themselves. They are generally classified into anionic, cationic, amphoteric and nonionic, depending on the charge of their headgroup. Among the groups listed above, the anionic surfactants are the most common in everyday and industrial uses and are toxic to both humans and the environment. In particular, anionic surfactants can bind to peptides, enzymes and DNA and alter their spatial layout (folding) and surface charge^5^. Such interactions can change the biological functions of biomolecules. Sodium dodecyl sulfate (SDS) is one of the most commonly used anionic surfactants, producing more than 3.8 million tons globally for industrial applications in cosmetics, clothing, food, fuel, and medicine^6^. Such mass production and use of SDS results in releases to the environment, with a semi-lethal concentration of not more than 45 μg/ml^7^ for algae, fish and crustaceans. In addition, it is known that surfactants can accumulate in the human body and cause autoimmune diseases, brain, liver, kidney and lung damage^8,9^. Besides the permissible limits for surfactants is 1 mg/l in water and at 0.5 mg/l for potable water^10^. In order to prevent negative environmental and human impacts of anionic surfactants (in particular SDS) in a timely manner, methods are needed to detect this surfactant in both wastewater and surface waters and in the soil^11^, food^9^, dust^12,13^, etc.

Spectrophotometric and potentiometric methods are the most common means of determining anionic surfactant, and chromatography is often used to concentrate and separate complex surfactant mixtures^14^. Most often, the ionometric determination of the surfactant is carried out using ionic electrodes, which makes it possible to determine the concentration of the substance under investigation in a short time (up to 30 min). However, this method has low sensitivity (280–600 μg/ml)^15^ and low selectivity, which does not allow the determination of surfactants in relatively complex samples. Spectrophotometric methods are also labour-free (10–30 min) and have a high sensitivity of 0.001 μg/ml^16,17^. The main disadvantage is the low specificity and dilution of the sample to the measuring limit of 0.01 μg/ml, which complicates the measurement procedure. These defects are corrected by chromatography, which allows separating the studied mixture and increasing the concentration of the required substance, but this procedure requires a minimum of several hours^15^.

There is therefore a need to develop a fast, low-cost method for determining anionic surfactant with high selectivity (specificity) that allows measurements to be made at environmentally toxic concentrations (10–50 μg/ml). Therefore, a quick semi-quantitative or qualitative determination of the substance by means of various rapid tests, such as paper tests, is sufficient for a number of practical tasks to determine the surfactants before applying a more precise and labour-intensive method systems, tracer powders, fabrics, polymer films, tablets^18–21^. In particular, Dmitrienko’s work with co-authors presents a method based on adsorption of a red-colored polyurethane foam (PUF) complex of an anionic surfactant with cation 1,10-fenantrolinate iron complex(II)^22^. This method allows the determination of anionic surfactants between 1 and 30 μg/ml. But all these systems have a common disadvantage—the need to use toxic reagents.

Thus, we propose a non-toxic diagnostic system based on polyelectrolyte microcapsules for quick, cheap and highly selective qualitative and semi-quantitative determination of SDS in the medium.

## Materials and methods

Polystyrene sulfonate sodium (PSS) and polyallylamine hydrochloride (PAH) with a molecular mass of 70 kDa Sigma (Merck KGaA, Darmstadt, Germany), fluorescein isothiocyanate (FITC) Sigma (Merck KGaA, Darmstadt, Germany); ethylenediaminetetraacetic acid (EDTA), calcium chloride (CaCl_2_ × 2H_2_O), sodium dodecyl sulfate, sodium chloride and sodium carbonate from Reahim (Reahim AO, St. Petersburg, Russian Federation) were used.

### Preparation of fluorescently labelled PAH

FITC was slowly added to a stirring (300–400 rpm) solution of polyelectrolyte (10 mg/ml) in 50 mM borate buffer, pH 9.0. The components were fused in a molar ratio of FITC: PAH = 1: 100. After that, its solution was incubated for 1.5–2 h. After incubation, the solution was dialyzed against water (10 l) overnight.

### Preparation of CaCO3 microspherolites

0.33 M Na_2_CO_3_ solution was rapidly added to the 0.33 M CaCl_2_ stirring solution^23^. The stirring was continued for 30 s. The suspension was maintained until complete precipitation of the formed particles. The process of “ripening” of microspherolites was controlled with the help of a light microscope. Then, the supernatant was decanted, the precipitate was washed with water and used to prepare PMC. The microparticles were obtained with an average diameter of 3 ± 1 µm. The size of particles was controlled by light microscopy and determined in the program ImageJ.

### Preparation of polyelectrolyte microcapsules

**Figure 1:**
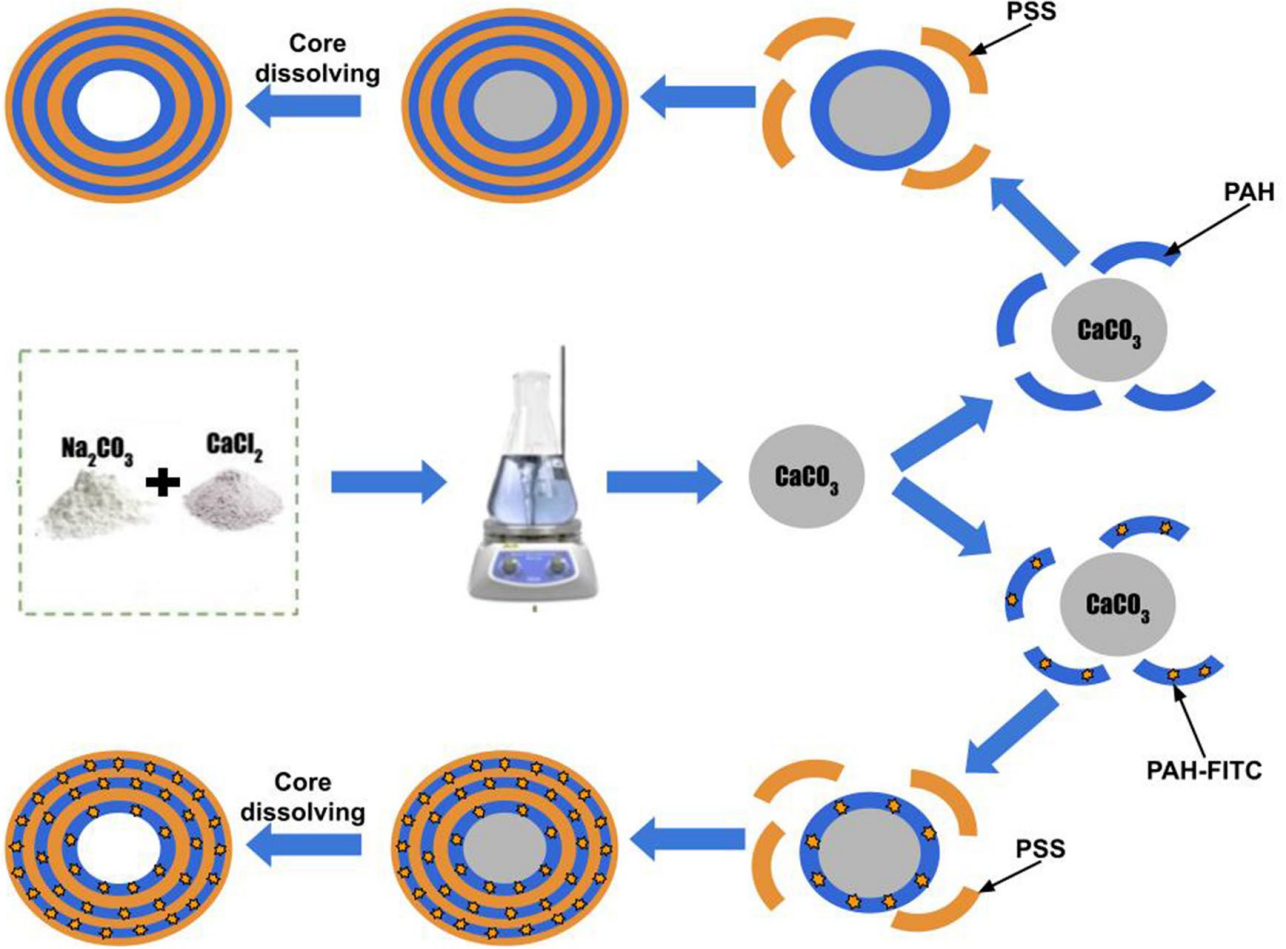
Stages of polyelectrolyte microcapsules preparation.

The polyelectrolyte microcapsules was obtained by alternately adsorbing the oppositely charged polyelectrolytes onto a dispersed microparticle (core), followed by dissolution of this cores. At the moment of dissolution of CaCO_3_ core the inner space of PMC is filled by interpolyelectrolyte complex 40. The microcapsule production process is shown in Fig. 1. Alternate adsorption of PSS and PAH on the surface of CaCO_3_ microspherolites was carried out in solutions of polyelectrolytes with a concentration of 2 mg/ml containing 0.5 M NaCl. Each step of adsorption was followed by a triple wash with a 0.5 M NaCl solution, which was necessary to remove unabsorbed polymer molecules. The particles were separated from the supernatant by centrifugation. After applying the required number of layers, the carbonate kernels were dissolved in a 0.2 M EDTA solution for 12 h. The resulting capsules were washed three times with water to remove core decay products. In the case of a qualitative method for SDS determination PMC contained FITC labelled PAH. The microcapsules were obtained with an average diameter of 3 ± 1 µm. The size of microcapsules was controlled by light microscopy and determined in the program ImageJ. The amount of the formed microcapsules was determined in the Goryaev chamber (40 × 106).

### Registration of an envelope dissociation of FITC-labelled PAH from polyelectrolyte capsules

A microcapsules envelope dissociation was analysed by fluorescent spectroscopy. The polyelectrolyte microcapsules containing FITC-labelled PAH in their envelopes and encapsulating FITC-labelled were centrifuged at 3000 rpm for one minute. Fluorescence of the supernatant was measured. The fluorescence spectra were registered with Cary Eclipse (USA) in a thermal controlled cuvette with 1 cm path length at light excitation with 273 nm wavelength. Each sample was measured three times.

### Registration of optical density of polyelectrolyte microcapsules

The suspension of PMC (4.1 × 10^8^ microcapsules) was added to a thermostated cuvette with an optical path length of 1 cm and measured spectrophotometrically at a wavelength of 450 nm. Each sample was measured three times.

### PMC incubation in SDS solution

Polyelectrolyte microcapsules were incubated in a solution of SDS of the required concentration with constant shaking on the vortex (500 rpm for the required time).

## Results and discussion

### Development of a qualitative method for SDS determination

**Figure 2:**
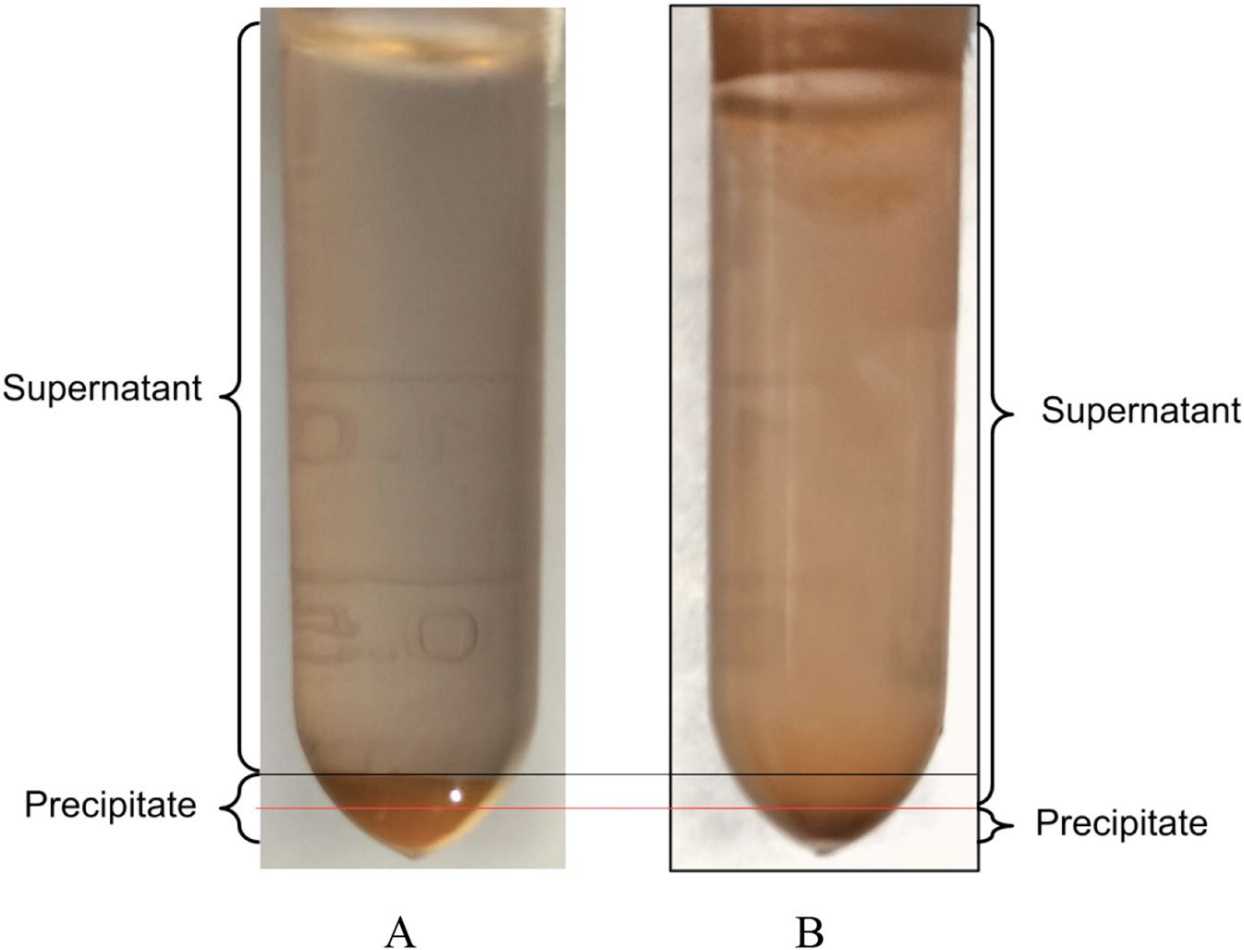
PMC precipitated suspensions incubated 20 min in distilled water (**A**) and in SDS solution 30 µg/ml (**B**).

We have found that polyelectrolyte microcapsules (PMC) of the (PAH/PSS)_3_PAH composition are destroyed in SDS solution. On Fig. 2 presents photographs of a precipitated suspension of PMC (120 million capsules, 3 ± 1 µm) that incubated 20 min in distilled water and in a solution SDS 30 µg/ml. The figure shows that the amount of deposited after incubation of PMC in solution SDS (B) is significantly lower than after the incubation of PMC in distilled water (A).

Based on this effect, we have proposed the use of PMC as a quality chemical method for determining SDS in water.

**Figure 3:**
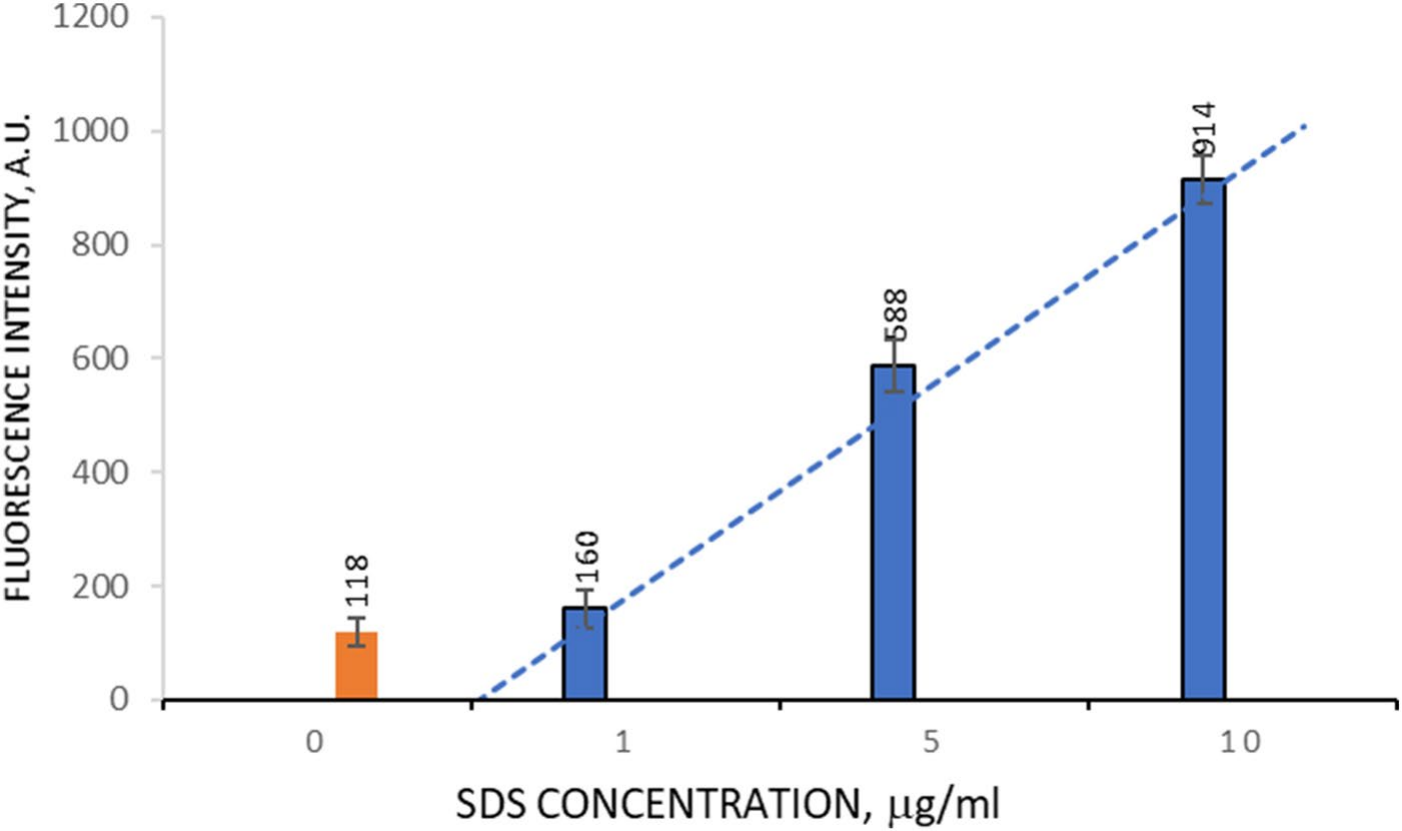
Fluorescence intensity of supernatant depending on SDS concentration (μg/ml).

A seven-layer PMC containing PAH covalently linked to FITC (PAH-FITC) in all even layers was prepared. The destruction of PMC (5 × 10^8^ microcapsules) containing PAH-FITC will result in the release of the tagged polyelectrolyte into the supernatant if SDS is present in solution. Fluorescence of labeled polyelectrolyte in supernatant will signal the presence of anionic surfactant in solution. The resulting microcapsules were incubated in SDS solutions with concentrations of 1, 5 and 10 μg/ml for 20 min. After depositing the microcapsules at 15,000 revolutions over 1 min, the fluorescence intensity of the supernatant was measured (Fig. 3).

From the figure it can be seen that significant differences in fluorescence intensity can be observed starting from a concentration of SDS of 5 μg/ml. It can therefore be concluded that the system detects the presence of SDS in water at concentrations of 5 μg/ml and above.

**Figure 4:**
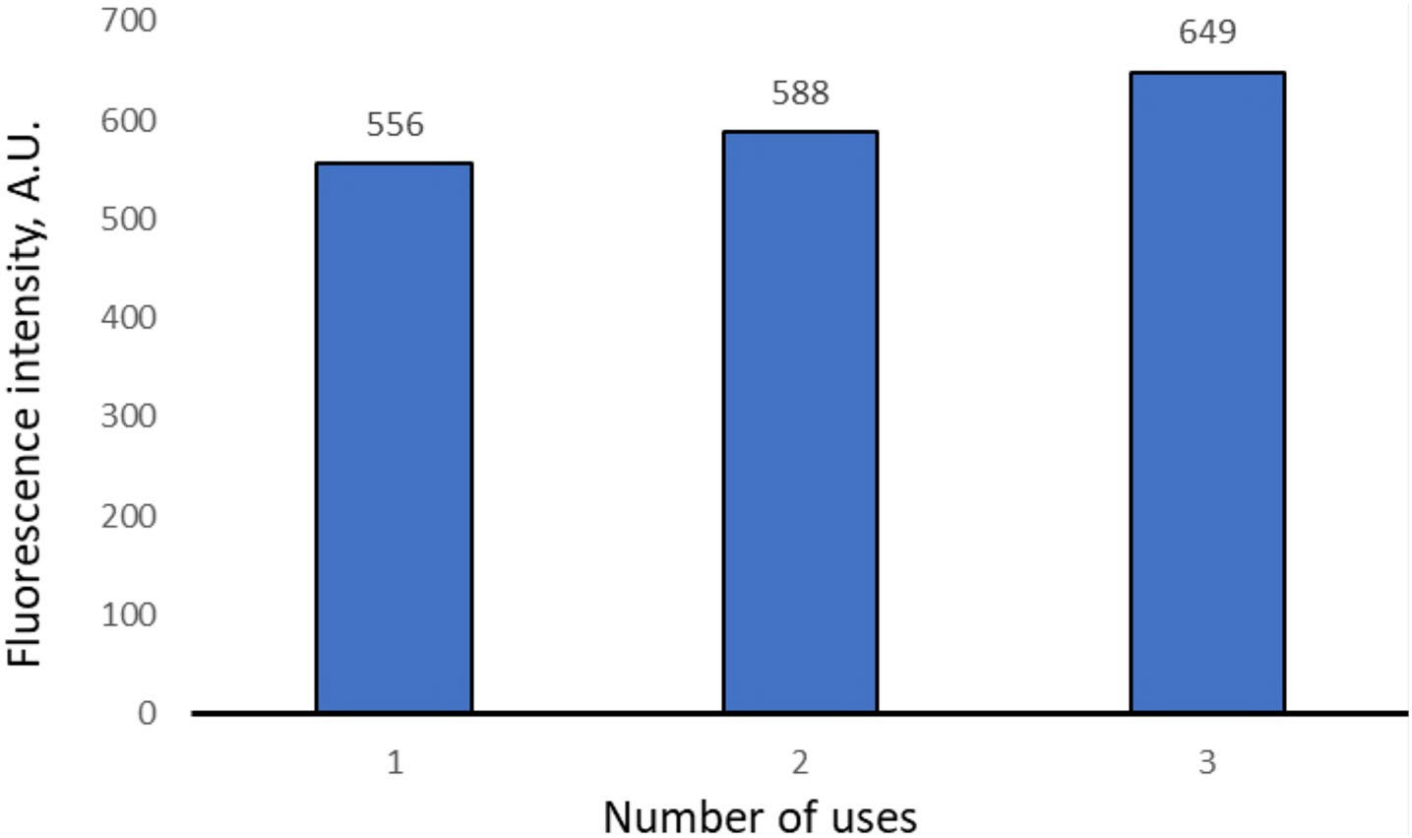
Fluorescence intensity of supernatant depending on the number of applications.

One possible advantage of a PMC-based diagnostic system is their reuse. Therefore, we have studied the reuse of PMC—(PAH-FITC/PSS)_3_PAH-FITC. For this purpose, 20 min were incubated PMC in a sample that contained SDS 5 μg/ml. After incubation of the suspension, the capsules were precipitated and removed from sample. The capsules were transported to the next sample containing a SDS solution of the same concentration. The intensity of fluorescence of supernatants of each sample was measured. The procedure was carried out three times and the results are presented in Fig. 4.

As shown in Fig. 4, the threefold use of the same PMC has little effect on fluorescence intensity, the standard deviation of fluorescence intensity of the supernatant does not exceed 8%.

According to the results obtained above, we propose to use the resulting system for *repeatedly qualitative determination* of SDS in solution with a concentration above 5 μg/ml, which corresponds to the fluorescence intensity of solution greater than 500 A.U.

### Development of a quantitative method for SDS determination

**Figure 5:**
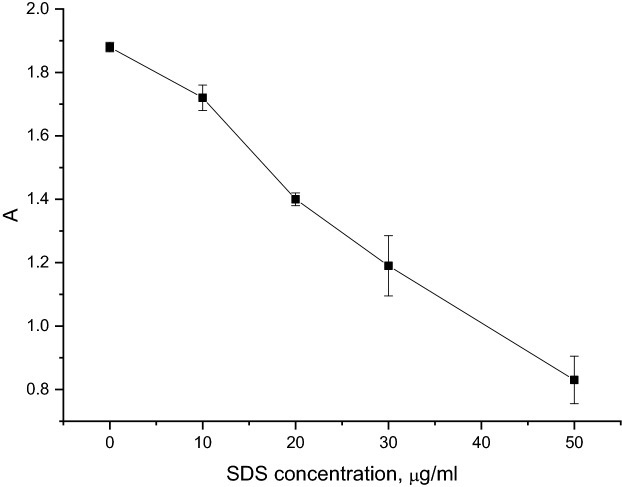
Light absorption of PMC (2 × 10^8^ microcapsules per ml) suspension by SDS concentration (μg/ml).

In order to detect higher SDS concentrations, we have proposed another system for recording the break-up of microcapsules. We tracked the decay of the capsules by the turbidity of their suspension before and after incubation in SDS solutions of various concentrations. The results of this study are presented in Fig. 5.

It can be seen from the figure that the turbidity of the solution declines linearly with the surfactant concentration rising from 10 to 50 μg/ml, with the standard deviation not exceeding 11%. This means that SDS can be determined in a given concentration range.

**Figure 6:**
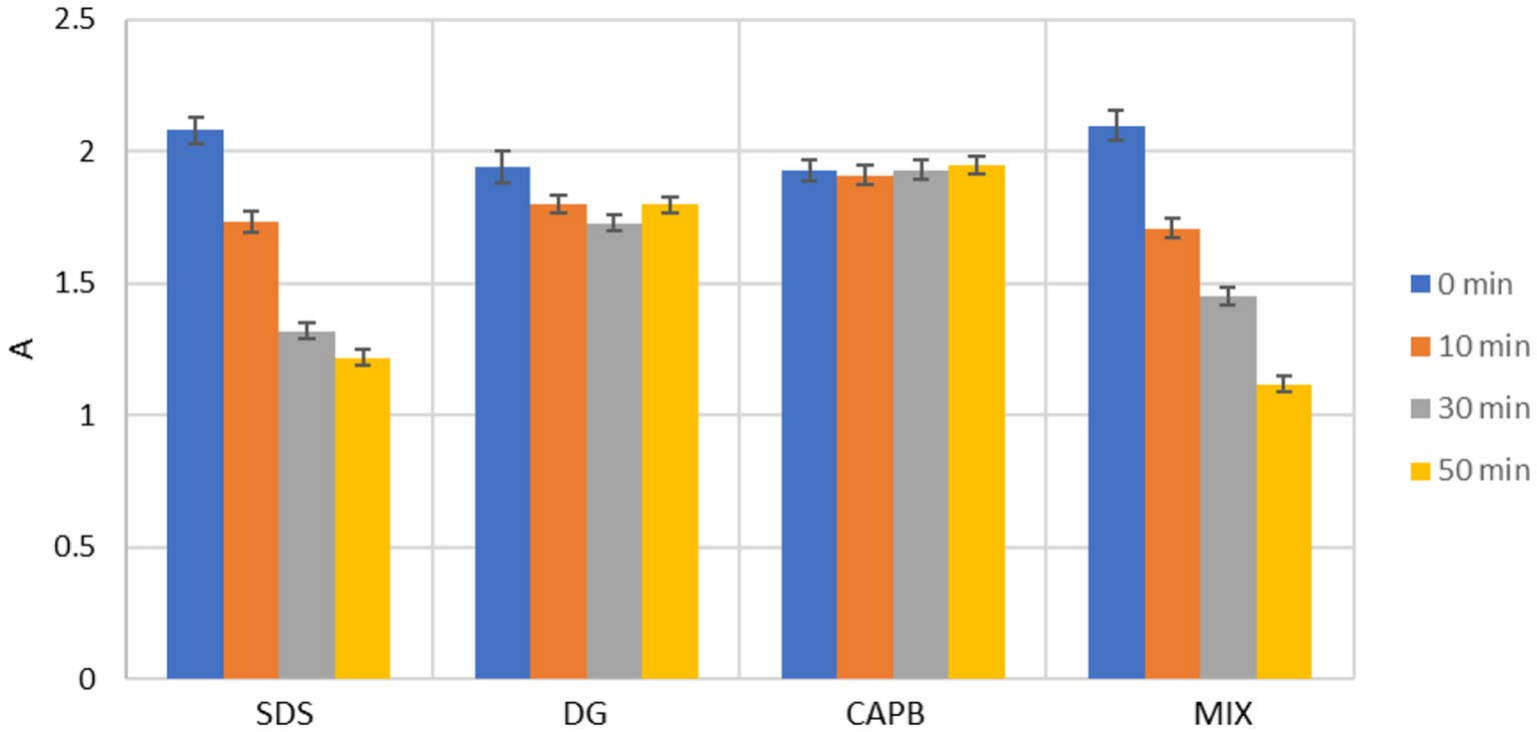
Light absorption of PMC suspension over time in the presence of various surfactants.

The next step in our research was to establish the specificity of our detection systems. For this, we studied the change of the optical density of a suspension of microcapsules over time in the presence of various surfactants: amphoteric surfactant—cocamidopropyl betaine (CAPB, 30 μg/ml); non-ionic surfactant—decyl glucoside (DG, μg/ml); anionic surfactant—sodium dodecyl sulfonate (SDS, μg/ml); a mixture of KAPB, DG and SDS (30 μg/ml each). The research results are shown in Fig. 6.

It can be seen from the Fig. 6 that the CAPB and DG did not affect to microcapsules stability during 50 min of incubation. Also, the surfactants (DG and CAPB) after mixing with SDS did not affect to destruction of PMC by SDS solution. From these data, it can be concluded that the detection systems we have proposed are specific to SDS and will not falsely trigger in the presence of other surfactants in the solution.

## Conclusion

It was discovered that polyelectrolyte microcapsules (PMC) of the composition (PAH/PSS)3PAH are destroyed in solution sodium dodecyl sulfate (SDS). And it was suggested that PMC be used as a quality chemical method to determine SDS in water. The resulting system detects the presence of SDS in water at concentrations of 5 μg/ml and above, with the possibility of repeated application at a standard deviation of not more than 8%.

In order to detect higher SDS concentrations, we have proposed a diagnostic system based on a change in the turbidity of the suspension before and after incubation in the solution of the anionic surfactant caused by the destruction of the PMC. The quantitative diagnostic system has a range of identifiable concentrations of SDS from 10 to 50 μg/ml, with a standard deviation of no more than 11%.
